# Supplementary material for: Perceived ease of use of Truenat among laboratory staff as a new diagnostic tool for TB in Nigeria: Result of a pilot roll-out
Source: PLOS Glob Public Health. 2024 Sep 20;4(9):e0003652. doi: 10.1371/journal.pgph.0003652 (PMC11414918; doi:10.1371/journal.pgph.0003652)
Supplement: Attachment — Submitted filename: Reviewer comments and response from Authors.docx [file pgph.0003652.s002.docx]

**Reviewer’s comments and response from Authors**

**Reviewer’s comment**

Reviewer #1: Line no. 226-228: Table 4 shows the power source to charge the Truenat machine. The major sources of power supply for charging the Truenat machine included public power supply, 65.8% and generators, 65.8%” Does this mean all the public power sources were using generators?

**Author response**

**The health facilities where the Truenat machines were situated were supported by NGOs who were implementing partners for the USAID-funded Introducing New Tools Project (iNTP). They included KNCV Tuberculosis Foundation Nigeria and IHV-Nigeria and as such for ease of service delivery such health facilities could have access to both public power supply and generators. Moreover, multiple responses to that variable by respondents were encouraged. What that means is that some of the health facilities have both public power supply and also generators.**

**Reviewer’s comment**

The two categories represented in Figure 3 are confusing. It would be better if the two responses were mentioned as “Good” and “Poor”

**Author response.**

**Thanks for the observation. This has been corrected.**

**Reviewer’s comment**

The study didn’t evaluate the performance and ease of use of Truenat in high burden settings. The average number of Truenat tests performed in the study centres was just 10, while most high-burden centres may receive 30 to 50 samples for TB diagnosis in a day. This aspect was not studied or included in the discussion.

**Authors response**

**The pilot implantation of Truenat for TB diagnosis ensured that Truenat machines were located in the peripheral centers with low infrastructure settings where there are no GeneXpert machines. It is important to point out that health facilities in Nigeria with GeneXpert machines at present could be considered as high burden centers. Even at that, the health facilities where the Truenat machines are located also receive samples from other peripheral centers around it. The emphasis as explained in the result is that perhaps due to the fact that Truenat machines in Nigeria at present could only take two samples at a time that the much the Lab staff could process within the eight hours designated for work in a day is ten samples.**

**Reviewer’s comment**

The ease of use of Truenat has been compared only with microscopy. What was the opinion of the respondents in comparison to Genexpert?

**Author response**

**In the introduction section of the manuscript, some comments/comparisons were made relating Truenat and GeneXpert machines. However, because Truenat was located in low infrastructure settings and hard to reach areas where the health workers have little or no experience with GeneXpert, comparison was made with sputum smear microscopy which the health workers were familiar with. The focus of this study was the Lab staff so comparison was made with what they were familiar with.**

**Reviewer’s comment**

Reviewer #2: The manuscript is well written and addresses one or the major challenges of performance of Truenat in programatic setting.

**Author response**

**Thank you for the kind words.**

**Reviewer’s comment**

Truenat Rif testing has more indeterminate, has the authors try to capture as part of the performance.

**Author response**

**Thanks for the comment.** However, exploring the factors associated with indeterminate test results with Truenat machine was outside the scope of the study

**Reviewer’s comment**
2. Performance of Truenat compared to smear may be less labour intensive but compare to GeneXpert its more labour intensive and the chances of cross contamination during the testing may be high with out training. This has not been discussed.

**Author response**

**The health workers were the focus of this study. In the pilot implementation of Truenat for TB diagnosis in Nigeria, the Truenat machines were located in peripheral centers with low infrastructure settings where there are no GeneXpert machines. The health workers were only familiar with sputum smear microscopy, hence the comparisons were made in that direction.**

**Reviewer comment**
3. The more common true prep issue of blockage is due to improper mucolysis or the machine problem has not been clearly marked in the manuscript.

**Author response**

**This was highlighted in line 231 - 232 in the results section. (Table 5). However, the exact causes were not explored in details.**
